# A Single Case Evaluation of an Emotion Regulation Training in Adolescents

**DOI:** 10.5334/pb.1337

**Published:** 2025-06-09

**Authors:** Elisa Boelens, Laura Wante, Leentje Vervoort, Sandra Verbeken, Lien Goossens, Caroline Braet

**Affiliations:** 1 Department of Developmental, Personality, and Social Psychology, Ghent University, Henri Dunantlaan 2, 9000 Ghent, Belgium; 2 Ghent University, Department of Developmental, Personality and Social Psychology, Henri Dunantlaan 2, 9000 Ghent, Belgium; 3 Radboud University, Behavioural Science Institute, Houtlaan 4, 6500 Nijmegen, The Netherlands

**Keywords:** emotion regulation, youth, psychopathology, training

## Abstract

The high prevalence of mental health problems, especially in adolescence, promotes research on psychological interventions. Recently, focus has shifted from disorder specific to transdiagnostic interventions. Since emotion regulation (ER) underlies various mental disorders, targeting this transdiagnostic mechanism is Particulary interesting. The current study aimed to investigate the feasibility and effectiveness of an emotion regulation training for children and adolescents (EuREKA) in six adolescents (*M* = 12.50, *SD* = 1.52; 50% girls) enrolled in an inpatient treatment center for internalizing and/or externalizing problems. Using a single case design, ER was monitored weekly during both baseline and treatment phase. In addition, pretraining and posttraining measurements on both psychopathology and ER were included. Although caution is warranted due to the nature of the study and the small sample size, the overall results suggest that EuREKA is a feasible and potentially promising approach for treating both internalizing and externalizing problems. However, some mixed results in the individual outcomes were observed, making it challenging to provide *general* conclusions on the effectiveness of EuREKA.

## Introduction

### Towards a Transdiagnostic Approach

Adolescence is a critical developmental phase in which 75% of all mental disorders manifest before the age of 24 (Wilson, 2020). Although current research is inconsistent in terms of determining the prevalence of adolescent mental health problems, one thing is not up for debate: over the past decade the numbers of both internalizing and externalizing disorders remained consistently high (i.e., ranging from 7.6% to 27,2%) with a recent peek during the COVID-19 pandemic (Kauhanen et al., 2022; Kovess-Masfety et al., 2016; Luijten et al., 2021; Waddell et al., 2014; Whitney & Peterson, 2019).

Simultaneously, perspectives on the classification, development and maintenance of psychopathology have shifted in the last decade (Wu et al., 2021). Moving forward from a categorical point of view, research is now more and more focused on a dimensional perspective such as the Hierarchical Taxonomy Of Psychopathology (HiTOP) and the Research Domain Criteria (RDoC) (Casey et al., 2013; Cuthbert, 2014; DeYoung et al., 2022; Insel et al., 2010; Kotov et al., 2018; Kotov et al., 2017). These approaches are more in line with clinical reality, as they provide a strong framework to research and to understanding for example comorbidity and symptom shifts. Especially with regard to comorbidity, youth rarely present themselves with one delineated problem raising the question on what may be the common ground between different disorders (Jacobi et al., 2014; Lehmann et al., 2013; Newman et al., 1996; Teplin et al., 2021).

This question has initiated research on transdiagnostic factors (i.e., factors underlying various types of psychopathology) (Dalgleish et al., 2020; Krueger & Eaton, 2015; Schaeuffele et al., 2021). One central transdiagnostic factor that is currently of high interest is *emotion regulation* (Fernandez et al., 2016).

### Emotion Regulation

Emotion regulation (ER) or ‘*the process by which individuals influence what emotions they have, when they have them, and how they experience and express them*’ (Gross, 1998, p. 275), has been implicated in a wide range of psychopathological problems. Research suggests that difficulties in ER contribute to approximately 75% of disorders including anxiety disorders, depressive disorders, conduct disorder, and eating disorders (Kring & Sloan, 2009).

To understand the processes of ER in all its facets, the integrated Adaptive Coping with Emotions model (ACE model, Berking & Whitley, 2014) was developed. In the ACE-model, ER is considered a situationally-dependent interplay between seven ER skills, namely: (1) being aware of the emotion, (2) identifying/labeling the emotion and interpreting body sensations related to the emotion, (3) unravelling the causes and maintenance of the emotion, (4) actively and adaptively modifying the emotion using specific strategies, (5) accepting the emotion when needed and tolerating it when it cannot be changed, (6) confronting and approaching the situation that causes distress and (7) supporting oneself when facing the situation and undesired emotion.

According to the ACE model, modification of negative emotions (4) and acceptance and tolerance of emotions (5) are the central ER skills and problems with these skills are directly related to psychopathology. The other skills, such as awareness (1) and effective self-support (7) are considered facilitators that promote the use of the two central ER skills (Aldao et al., 2016; Berking & Lukas, 2015).

### Emotion Regulation Interventions

Based on the ACE model the Affect Regulation Training (ART) was developed to improve adaptive ER in adults with a wide range of clinical problems (Berking et al., 2008; Berking & Lukas, 2015; Berking & Schwarz, 2014). The ART is founded on the ACE model and includes seven modules targeting various ER skills. The techniques employed are derived from cognitive behavioral therapy, acceptance and commitment therapy, emotion-focused therapy, solution-focused therapy and positive therapy. ART has been evaluated multiple times in both convenient samples (i.e., adults with a stressful occupation) and clinical samples (i.e., adults with depression) and proven to be effective in both improving ER skills and mental well-being (Berking et al., 2008; Berking et al., 2013; Berking et al., 2010).

### Emotion Regulation Interventions in Youth

Adolescence is a period of heightened demands on ER processes. Cracco et al. (2017) found evidence for a maladaptive shift in the application of adaptive and maladaptive ER strategies. In literature adaptive ER strategies are described as strategies that lead to *adequate ER* as they reduce negative affect and have the possibility to increase positive affect. Moreover, they have been associated with a greater level of psychological well-being in both youth and adult samples (Aldao et al., 2014; Cicchetti et al., 1995; Werner & Gross, 2010). Examples of well-studied adaptive ER strategies are cognitive reappraisal, problem solving and distraction (Gross, 1999). In contrast, research shows that maladaptive ER strategies may in the short term lead to brief positive effects on experienced emotions but have been linked to persistent negative affect and emotional problems in the long term in both adults and youth (Aldao & Nolen-Hoeksma, 2010; Werner & Gross, 2010). Examples of maladaptive ER strategies that have been extensively researched are rumination and suppression (Aldao & Nolen-Hoeksma, 2010; Dryman & Heimberg, 2018; Moore et al., 2008).

Around the age of 12 to 15 years, during the maladaptive shift, research suggests the use of adaptive ER strategies decreases, and the use of maladaptive ER strategies increases compared to children and adolescents in younger and older age groups. This maladaptive shift provides a potential explanation for the spike in psychological problems (Cracco et al., 2017; Zimmermann & Iwanski, 2014) and emphasizes the need for specific attention for ER training within this age group.

Based on both the ART for adults (Berking & Lukas, 2015) and research evaluating the effects of the separate ER skills included in the ACE model in children and adolescents (Boelens et al., 2022; Volkaert et al., 2020; Wante et al., 2018), the Emotion Regulation Training for Children and Adolescents (EuREKA) was developed (Braet & Berking, 2019; Debeuf et al., 2020; Verbeken et al., 2019), for children and adolescents between 10 and 16 years old. The training is a child-friendly version of the ART and focuses on training facilitating ER skills (i.e., emotional awareness) and specific ER strategies (e.g., problem solving) in a fixed sequence (see procedure below; Verbeken et al., 2019). EuREKA is designed as a transdiagnostic ER training, both in a prevention and an intervention format.

Currently, the EuREKA *prevention* protocol has been evaluated in a randomized controlled trial (RCT) in community youth (aged 12–13) going through the transition from primary to secondary school (Volkaert et al., 2018). The school-based training was evaluated as feasible and showed significant improvements on depressive symptoms, self-esteem and indirect bullying immediately posttreatment compared to youth who did not follow the program (Volkaert et al., 2022).

Furthermore, the EuREKA *intervention* protocol has been evaluated in an RCT in children and adolescents with obesity enrolled in an inpatient multidisciplinary obesity treatment program. In this specific sample, the EuREKA intervention resulted in an increase in several ER skills (i.e., emotional awareness, problem solving and evoking a positive mood) posttreatment and effective self-support at 3-month follow-up compared to a control group of youth within the same treatment center only receiving TAU (Boelens et al., 2022; Debeuf et al., 2022).

## Current study

Although the first studies on EuREKA show promising results in community and at-risk youth samples, no studies so far evaluated EuREKA in clinical groups of youth referred specifically for internalizing and/or externalizing problems (Debeuf et al., 2020; Debeuf et al., 2022; Volkaert et al., 2022). Therefore, the current study aims to investigate the **feasibility** and **effectiveness** of EuREKA as a group format in adolescents with internalizing and/or externalizing problems. The present study will examine the feasibility of Eureka in a sample of clinical youth based on different parameters (e.g. satisfaction, acceptability). Moreover, during weekly supervision with trainers, treatment integrity is evaluated. Intervention outcomes will be evaluated using a single case design (Maric & van der Werff, 2020). First, weekly changes in ER skills are investigated for each adolescent from baseline to the end of training. Second, pretraining to post training changes in ER and psychopathology are evaluated on an individual and a group level.

It is expected that (a) EuREKA will be feasible on top of TAU, (b) adolescents will have more and better general and specific ER skills *during* EuREKA and (c) adolescents will have more and better general and specific ER skills and will experience less internalizing and/or externalizing problems *at the end* of EuREKA.

## Method

### Participants and Trainers

The current study included data from adolescents and trainers. Six adolescents between 11 and 15 years old (*M* = 12.50, *SD* = 1.52; 50% girls) participated in the study. All adolescents were patients in an inpatient multi-functional treatment center (MFC) for adolescents with behavioral (i.e., externalizing) and/or emotional (i.e., internalizing) problems (see Table 1 for a detailed overview). Two similar MFC’s participated in the current study. Adolescents were included when they (a) were between 11 and 15 years old, (b) had not yet received any ER training, (c) were evaluated as eligible through clinical observations by the psychologist of the treatment center and (d) had a normal intelligence. Treatment as usual (TAU) in the center involves weekly individual sessions mainly focused on pedagogical and basic psychological support (e.g., personal goals, following rules and agreements within the center, anger management, behavioral activation). On top of TAU, participants completed the EuREKA training delivered in a group format. The EuREKA training was administered by two of four trainers. The trainers were female students in the final year of their master in clinical psychology and were 23 years old. Prior to the study, they were trained in the EuREKA protocol and during the study they participated in weekly supervision meetings with the first author.

**Table 1:** Detailed overview of participants.


PARTICIPANT	AGE	GENDER	TREATMENT CENTER	DSM SCALES (ASEBA-YSR)

1	15	Girl	MFC for youth with behavioral and emotional problems (A)	**Affective** problems (subclinical), **Anxiety** problems (clinical), **ADHD** (clinical), **Conduct** problems (subclinical)

2	13	Boy	MFC for youth with behavioral and emotional problems (A)	**Affective** problems (clinical), **Anxiety** problems (clinical), **Somatic** problems (subclinical)

3	11	Boy	MFC for youth with behavioral and emotional problems (A)	**Affective** problems (clinical), **Anxiety** problems (clinical), **Conduct** problems (subclinical)

4	12	Boy	MFC for youth with behavioral and emotional problems (B)	No (sub)clinical scores Highest score on **ADHD**

5	13	Girl	MFC for youth with behavioral and emotional problems (B)	**Affective** problems (subclinical), **ODD** (clinical), **Conduct** problems (subclinical)

6	11	Girl	MFC for youth with behavioral and emotional problems (B)	**Affective** problems (subclinical), **Anxiety** problems (clinical), **Somatic** problems (clinical), **ADHD** (subclinical)


*Note*. Two different multi-functional centers (MFC) with similar programs are designated as (A) and (B); Achenbach System of Empirically Based Assessment (ASEBA); Youth Self-Report (YSR), T-scores on DSM-oriented are categorized as <65 = normal, 65–69 = subclinical, ≥70 = clinical; see also Measurements).

The study was submitted to and approved by the ethics committee of the faculty of psychology and psychological sciences (2020/58; Ghent University). Caregivers and trainers gave written active consent and adolescents gave written assent. After participating in the study, adolescents received a gift voucher for an online webstore.

### Measurements

#### Internalizing/Externalizing problems

**Achenbach System of Empirically Based Assessment (ASEBA)** includes questionnaires for indicating emotional and behavioral problems in children and adolescents (Achenbach & Verhulst, 2010). In the current study both the **Youth Self Report** (YSR) for adolescents between 11 and 18 years old and **The Child Behavior Checklist** (CBCL; caregiver report) were used (Achenbach & Edelbrock, 1991; Verhulst et al., 1997). The YSR contains 112 items and the CBCL 118 items. Two overarching dimensional subscales “Internalizing problems” and “Externalizing problems” can be calculated. Further, it is possible to calculate scores on six DSM scales: 1) affective problems, 2) anxiety problems, 3) pervasive developmental problems, 4) attention deficit/hyperactivity problems and 5) oppositional defiant problems. In addition, six norm-referenced DSM-Oriented Scales can be calculated including Affective Problems, Anxiety Problems, Somatic Problems, Attention Deficit/Hyperactivity Problems, Oppositional Defiant Problems, and Conduct Problems. Depending on the T-score and relative to the norm group, scores can be interpreted as normal, subclinical or clinical. Previous research shows good reliability and validity for both the YSR and CBCL (Achenbach & Edelbrock, 1991).

The **Children’s Depression Inventory** (CDI; (Timbremont et al., 2002) is used to screen for cognitive, affective and behavioral symptoms of depression in children and adolescents (age 8–21). The self-report questionnaire consists of 27 items. The total score gives an indication of the severity of depressive symptoms with a score higher than 13 indicating a high chance of depression. The Dutch version of the CDI has good internal consistency and moderate test-retest reliability (Timbremont et al., 2002).

#### Emotion regulation

The **Emotion Regulation Skills Questionnaire Junior (ERSQ-J)** (Van Beveren et al., 2024) was administered on a weekly basis. The ERSQ-J is a self-report questionnaire to assess ER skills (e.g., emotional awareness, effective self-support, modification of emotion, acceptance of emotions) and is based on the adult Emotion-Regulation Skills Questionnaire (ERSQ; (Berking & Znoj, 2008). It consists of 27 items answered on a five-point Likert scale ranging from 0 (= not at all) to 4 for (= almost always). For example: *“During the past week, I was aware of my emotions”*. Research on the Dutch version of the ERSQ-J shows good internal reliability for both the full questionnaire and the subscales in a community youth sample. Furthermore, good convergent validity was observed (Den Aantrekker et al., 2020).

The **Questionnaire for Emotion Regulation Strategies in Children and Adolescents** (FEEL-KJ; (Cracco et al., 2015; Grob & Smolenski, 2005)) assesses how children and adolescents between 8- and 18-years old cope with feelings of anger, sadness, and anxiety. In the current study, both the self-report and caregiver-report version of the FEEL-KJ were used. A total score can be calculated for adaptive, maladaptive, and external ER strategies. The FEEL-KJ is considered reliable and valid with an acceptable to good internal consistency across all subscales (Cronbach’s alpha between .64 and .94). Furthermore, an acceptable to good test-retest reliability was reported (Cracco et al., 2015).

#### Feasibility

##### Participants

Participants filled out a brief feasibility questionnaire (i.e., session rating scale) at the end of each session which was based on the “Barriers-to-Treatment-Participation Scale” (Kazdin et al., 1997) (see Table 2). This questionnaire included 6 statements regarding the session (see Table 2). The items were scored on a 5-point Likert Scale from 1 (= strongly disagree) to 5 (= strongly agree). Participants were also asked how they would rate this session overall on a scale from 0 (= totally not satisfied) to 10 (= very satisfied).

**Table 2:** Feasibility overall training.


QUESTIONS

I am satisfied with this session I felt like the trainer understood me The trainer listened to me I understood the information that was given The worksheets were helpful to work with What we did and what we talked about was important to me


*Note*. Based on the “Barriers-to-Treatment-Participation Scale”

##### Trainers

To check treatment integrity and collect feedback, qualitative data were collected from the trainers. They were invited to reflect on their own therapeutic actions during the training and provide content-related feedback on each session. All feedback was provided verbally during the weekly supervision session with the researcher (Nezu et al., 2008; Perepletchikova et al., 2007). All answers were meticulously written down and processed by the first author.

### Design

#### Single-case design

Single case designs require; (a) a baseline and a training phase, (b) data-collection at well-defined and preferable multiple time points during both phases and (c) no control group to investigate intervention effects. As the name implies, the focus is on change within individuals (single cases) rather than within a (large) group. This approach provides more flexibility for clinical scientists as fewer participants are needed. Although single-case designs cannot fully rule out alternative explanations, such as the effects of time or external influences, previous research shows that potential causal relationships can be examined (Barlow & Nock, 2009; Smith, 2012; Tarlow et al., 2020).

In the present study a **single-case AB design** was used (Maric & van der Werff, 2020) with (A) a baseline phase (i.e., where adolescents received TAU) and (B) a treatment/training phase. Participants were allocated to a predetermined baseline period of 3, 5 or 7 weeks (Spuij et al., 2013). By using these multiple datapoints, and monitoring participants for at least 3 weeks, it is possible to account for time or maturity effects and obtain a reliable pretraining measurement of the primary outcome (Jarrett & Ollendick, 2012; Maric & van der Werff, 2020). Furthermore, it is possible to examine how participants’ symptoms evolve during the baseline and treatment/training phase and whether there is visible (i.e., through visual analyses) improvement during and after the treatment/training phase.

### Procedure

#### Recruitment

Two youth inpatient treatment centers for emotional and behavioral problems participated in the current study. The adolescents who were eligible for the study and their caregiver were informed by the center’s psychologist(s) and received an information letter. Next, caregivers gave written active consent and adolescents gave written assent.

#### Assessment prior to and during EuREKA

**Figure 1:**
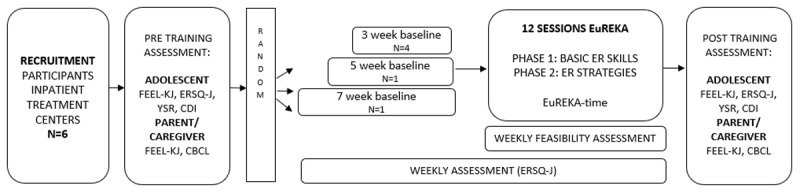
Study design.

At the start of the study (see Figure 1) pretraining assessment took place (duration 2h). Both adolescents and their caregiver (i.e., a parent or educator of the treatment center depending on the closest relationship) filled out questionnaires on psychopathology and ER via an online platform. Subsequently, participants started in the baseline phase and were asked to fill out a weekly ER questionnaire during the baseline phase.

Immediately after the baseline phase, the treatment/training phase (i.e., 12 weekly sessions EuREKA) started. At the start of each session, weekly assessment (i.e., ER questionnaire) took place and at the end the session rating scale with regard to feasibility was filled out by the adolescents.

#### Practical organization of EuREKA

Two groups of three adolescents were given the EuREKA training on a weekly basis, on top of ongoing TAU. EuREKA sessions were provided by two trainers (i.e., master students clinical psychology). Students followed a 1,5-day workshop on the EuREKA to become an EuREKA-trainer. Trainers were also invited to weekly supervision sessions with the researcher in which they were able to reflect on their functioning and discuss the content and the process of the prior and upcoming EuREKA session.

#### General information on EuREKA

**Figure 2:**
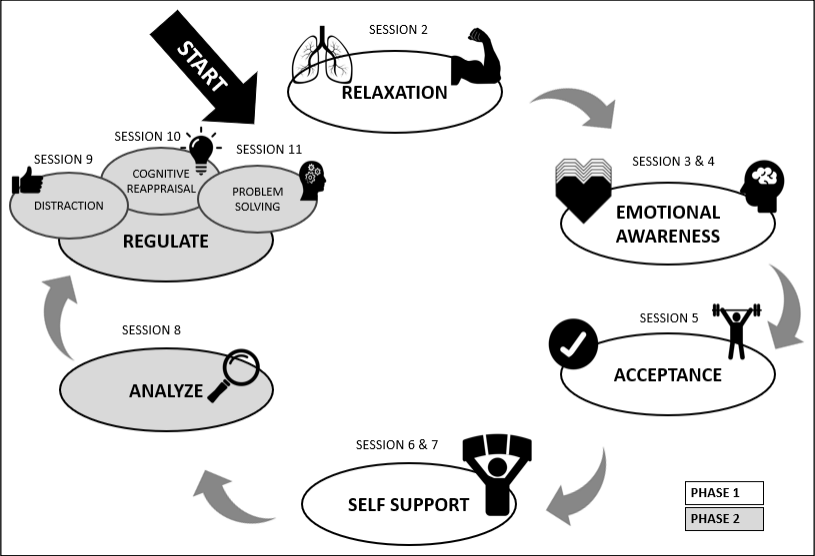
EuREKA-training/EuREKA-circle.

EuREKA consists of 12 sessions of one and a half hours. EuREKA aims to improve ER skills on top of TAU. The EuREKA program can be divided into 2 phases (see Figure 2). The first phase (session 2–7) is focused on training **ER facilitating skills** (e.g., relaxation; emotional awareness) and the second phase (session 8–11) is focused on training **specific ER strategies** (e.g., cognitive reappraisal, problem solving). Each session has a fixed structure to promote predictability and safety. More specifically, every session starts with a short recapitulation of the previous session and a discussion regarding the homework assignment (see EuREKA-time). Next, the content of the current session is introduced after which new skills are trained using psychoeducation and exercises. At the end of each session participants are informed about the upcoming homework assignments and receive a little incentive for their cooperation and motivation during the session (e.g., stress ball, emoji key chain).

#### Content of EuREKA

A detailed description of the EuREKA protocol can be found in the EuREKA handbook and published protocol paper (Braet & Berking, 2019; Verbeken et al., 2019, Debeuf et al., 2020). Specific exercises are published in the EuREKA workbook (Boelens et al., 2019).

#### Homework assignments during EuREKA

Between training sessions, participants were invited to complete homework assignments (i.e., EuREKA-time). EuREKA-time consists of an audio tape with instructions to further exercise the learned steps of de EuREKA-circle.

## Data-analysis

First, descriptive statistics regarding feasibility were obtained. On the weekly feasibility questionnaire, participants had the option to (strongly) agree or disagree with six questions regarding EuREKA, but were also able to give a neutral score (i.e., 0 = strongly disagree, 2 = disagree, 3 = neutral, 4 = agree, 5 = strongly agree). Therefore, it was determined a priori that criteria were deemed to be feasible if (on average) participants scored 3.5 or higher meaning that they agreed to totally agreed with the item. On the contrary, items with a score lower than 2.5 were categorized as treatment barriers as participants did (totally) not agree with these items (Koch et al., 2020; Thabane et al., 2016). Scores higher than 2.5 and lower than 3.5 are categorized as neutral.

In addition, overall ratings of each session were visualized using a line chart (0–10). A priori it was determined that scores between 0 and 2.5 were categorized as totally not satisfied, between 2.5 and 5 as not satisfied, between 5 and 7.5 as moderately satisfied and between 7.5 and 10 as very satisfied with the session. Lastly, qualitative analyses were carried out to explore treatment integrity and general feedback. Trainers’ responses were systematically coded, and themes were identified based on Braun and Clarke’s (2006) six-step approach. The analysis was conducted inductively, allowing themes to emerge from the data. To enhance rigor and credibility, peer debriefing was used where an independent researcher reviewed and discussed the coding and theme identification.

Second, regarding individual participant scores (a) weekly assessments were visualized using a line chart and (b), reliable change indexes ( \[\textstyle{RCI = {{{x_{2}-x_{1}}} \over {S_{diff}}}}\]) between all pretraining and posttraining outcomes were calculated to interpret training effects in terms of clinically significant change (Jacobson & Truax, 1992). In order for a change to be seen as statistically reliable at the p = . 05 level, RCIs must reach the level of 1.96. RCIs between 1.30 and 1.96 are considered a positive trend.

Third, to compare pretraining to post training group scores across all participants, a Wilcoxon Signed-Rank Test was used. In addition, effect sizes were calculated using the rank-based effect size (r), where values of 0.1 indicate a small effect, 0.3 a medium effect, and 0.5 a large effect (Conover, 1999).

## Results

### Feasibility

#### Adolescents

The average scores on group level (Table 3) indicate that participants agreed with the statement that the content of the sessions, interactions with the trainer and the used materials were useful/good and that they did not experience any significant treatment barriers (all scores > 3.5). Also, the individual results per participant indicate there were no treatment barriers (no scores <2.5). Nonetheless, participants 3, 5 and 6 gave a neutral response for two or more items (scores between 2.5 and 3.5).

**Table 3:** Feasibility evaluated by adolescents.


	PARTICIPANT 1			PARTICIPANT 2			PARTICIPANT 3			PARTICIPANT 4			PARTICIPANT 5			PARTICIPANT 6			OVERALL SCORES	
						
	M	MIN-MAX	SD	M	MIN-MAX	SD	M	MIN-MAX	SD	M	MIN-MAX	SD	M	MIN-MAX	SD	M	MIN-MAX	SD	M	SD

1. I am satisfied with the session	4.17	4–5	.39	4.50	3–5	.67	3.64	3–5	.92	3.82	1–5	1.40	3.18	3–5	.60	3.27	1–5	1.10	**3.9**	**.50**

2. I felt like the trainer understood me	4.41	4–5	.51	4.67	4–5	.49	3.46	3–5	.82	4.18	2–5	1.08	3.81	3–5	.60	3.18	1–5	1.32	**4.11**	**.48**

3. The trainer listened to me	4.58	4–5	.51	3.58	4–5	.51	3.63	3–5	.81	4.09	1–5	1.22	3.81	3–5	.60	3.18	1–5	1.33	**3.99**	**.55**

4. I understood the information that was given	4.41	4–5	.51	4.17	2–5	.94	3.18	3–5	.60	4	1–5	1.41	3.82	3–5	.75	3.36	2–5	.92	**3.82**	**.47**

5. The worksheets were helpful to work with	4.01	4–5	.29	3.75	3–5	.75	3.36	3–5	.81	4	1–5	1.41	3.36	2–5	.81	3.45	3–5	.82	**3.67**	**.32**

6. What we did and what we talked about was important to me	4.17	4–5	.39	4.10	2–5	1.00	3.45	2–5	.1.04	3.63	1–5	1.36	3.81	3–5	.63	3.45	3–5	.82	**3.84**	**.37**


*Note*. Strongly disagree (1), disagree (2), neutral (3), agree (4), strongly agree (5).

Participants were also able to give an overall score out of 10 for each session. On average for each session and across participants, scores ranged between 7.36 and 9.7 indicating that participants were moderately to very satisfied with the training. In addition, participants 1, 3 and 5 indicated to be very satisfied with all sessions. Participant 2 was moderately to very satisfied with the sessions. In the scores of participants 4 and 6, substantial variation was observed with evaluations ranging between totally not satisfied to very satisfied with the session.

#### Trainers

Feedback during the weekly supervision sessions covered five overarching themes (1) Treatment integrity and self-efficacy; (2) structural feedback on EuREKA, (3) session specific feedback, (4) external factors, (4) and (5) research related feedback.

With regard to **treatment integrity and self-efficacy**, all trainers felt capable and comfortable with the detailed training protocol. They reported that the protocol was easy to use, hands-on and feasible in the current treatment groups. In addition, trainers indicated that they were able to reach each predetermined ER goal. However, adding extra visual material or exercises would have facilitated the sessions that are cognitively demanding.

Next, **structural feedback on EuREKA** revealed several challenges. For example, trainers often experienced time pressure with 90-minute sessions being too short to finish all exercises. However, this was mostly linked to the fact that the weekly questionnaires and session ratings were also completed within the session and took 10–15 minutes. Next, three trainers evaluated the fixed introduction of each EuREKA session as too long and boring and indicated that this can be done in a more fun and efficient way. In addition, suggestions were made to provide more variation in the exercises (i.e., more creative techniques, more physical exercises, active movement) as it might make the training less monotonous. Furthermore, two trainers reported that although several strategies are trained during EuREKA, too little focus was put on emotional flexibility. Lastly, all trainers reported that the paper-and-pencil format of the homework assignment ‘EuREKA time’ is outdated and that an online alternative should be provided in the future. All trainers noticed that the motivation for the homework assignment decreased throughout the weeks and that not any adolescent succeeded in completing all weekly homework assignments.

Third, as part of the **session specific feedback**, three trainers indicated that the progressive muscle relaxation exercise in the second session lasted too long. Similar feedback was given for session four in which adolescents had to listen to several mindfulness exercises which was experienced as too monotonous and exhausting. For session seven, two of the trainers noted that “the empty chair exercise” required considerable (meta) perspective-taking and was too complex for some of the adolescents.

Fourth, two **external factors** influencing the training process were also noticed. First, as the sessions were mainly verbal and cognitive rather than visual and creative, all trainers reported that some adolescents had difficulties with sustained attention. Some of the adolescents started to lose focus or had difficulties with sitting down on their chair. Second, trainers reported also that the group atmosphere (i.e., getting along, feeling safe, respecting each other) was seen as crucial for the quality of the session and when tension between group members was present.

Finally, regarding the **research related feedback** trainers were unanimous. The ER questionnaire (start of the session) and feasibility questionnaire (end of the session) asked a lot of the participants and interrupted the flow of the sessions. The possibility for the trainers to have supervision sessions was evaluated as an added value and increased the quality of the training execution.

### Individual outcomes

Figures 3a, 3b, 3c, 3d, 3e, 3f present weekly scores on the ERSQ-J during the baseline phase (A) and treatment phase (B). Baseline scores show low initial values for four out of six youth. More specifically, two participants (PP5 and PP6) score ≤50 and two participants (PP1 and PP2) score ≤60 leaving room for improvement. Only participant 4 (PP4) scores consistently above 60 at baseline. Individual outcomes on the weekly ERSQ-J questionnaire showed pretraining to post training changes of +22%, –20%, +10%; –40%, +71%, +66% that will be discussed for each participant below.

**Figure 3a:**
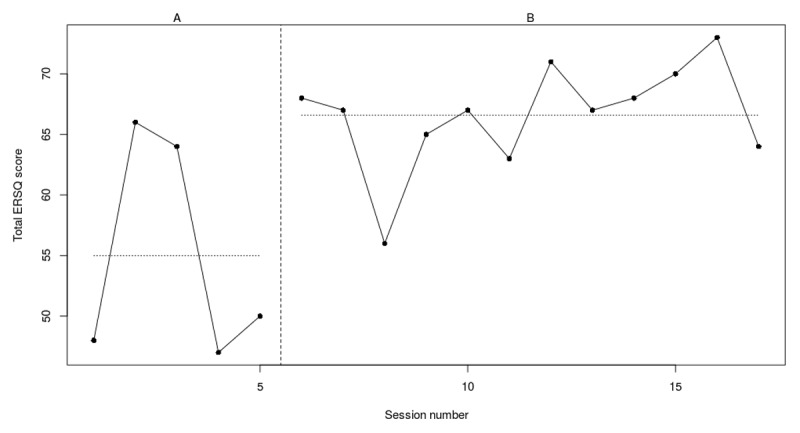
Weekly scores on the ERSQ-J during the baseline phase (A) and treatment phase (B) for PP01. *Note*. PP = participant.

**Figure 3b:**
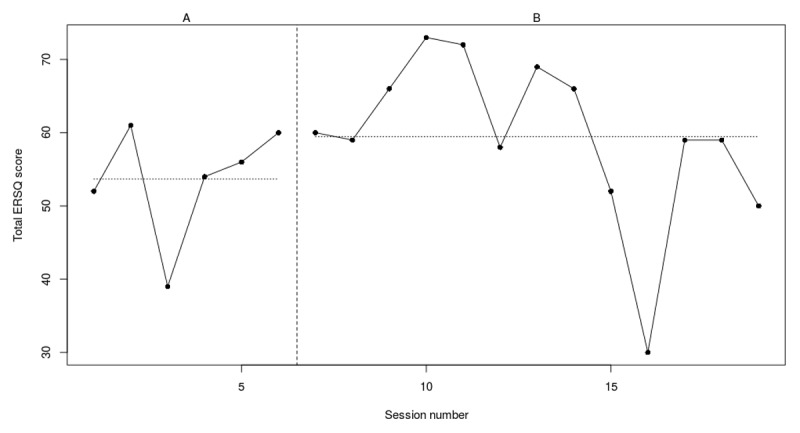
Weekly scores on the ERSQ-J during the baseline phase (A) and treatment phase (B) for PP02. *Note*. PP = participant.

**Figure 3c:**
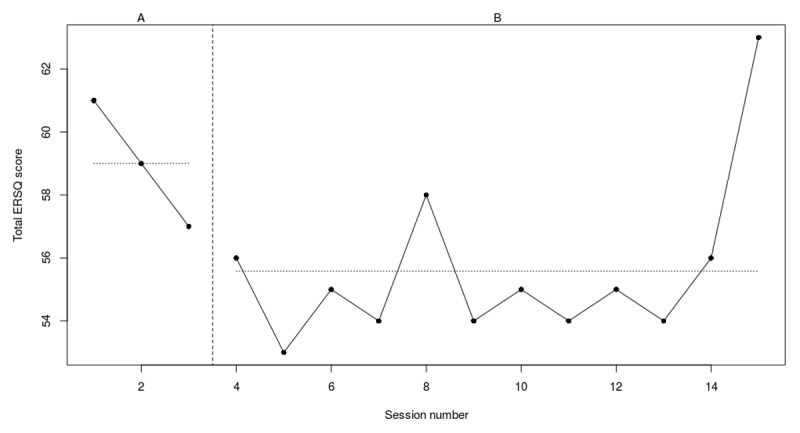
Weekly scores on the ERSQ-J during the baseline phase (A) and treatment phase (B) for PP03. *Note*. PP = participant.

**Figure 3d:**
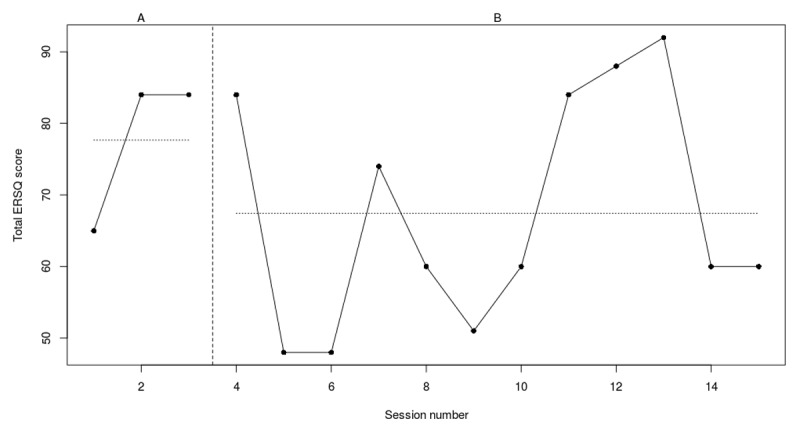
Weekly scores on the ERSQ-J during the baseline phase (A) and treatment phase (B) for PP04. *Note*. PP = participant.

**Figure 3e:**
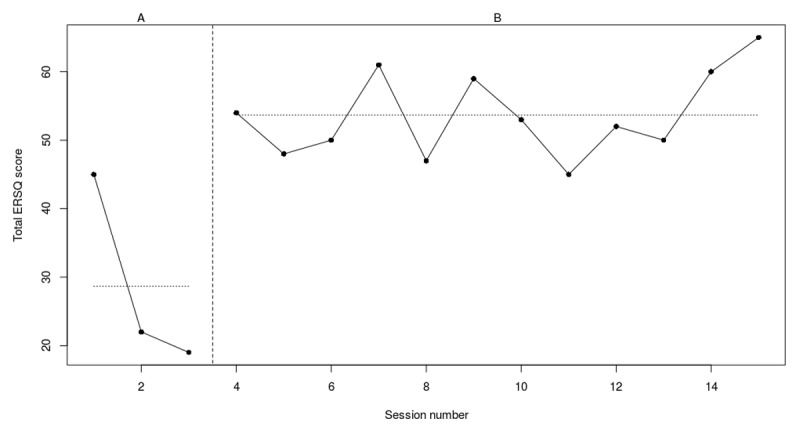
Weekly scores on the ERSQ-J during the baseline phase (A) and treatment phase (B) for PP05. *Note*. PP = participant.

**Figure 3f:**
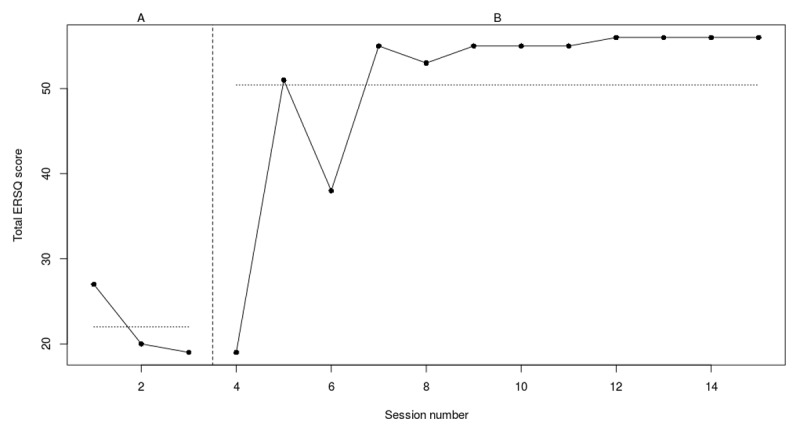
Weekly scores on the ERSQ-J during the baseline phase (A) and treatment phase (B) for PP06. *Note*. PP = participant.

#### Participant 1

During the 5-week baseline, participant 1 started with a score of 48 on the ERSQ-J after which a sudden increase (+27%) was noticed. At the end of the baseline scores stabilized to 50 and increased to 64 (+22%) at the end of treatment. During training (see Figure 3a), scores immediately increased, with the highest scores in the second phase of the training (i.e., training specific ER strategies). Moreover, looking at the RCIs (see Table 4), there was a significant increase for the Feel-KJ adaptive strategies self-report (SR) and caregiver-report (CR) and a significant decrease for the Feel-KJ maladaptive strategies (CR) and both the CBCL internalizing and externalizing problems. No significant results were found for the CDI, Feel-KJ maladaptive strategies (SR) or YSR internalizing and externalizing problems.

**Table 4:** Primary outcomes at pretraining and post training for each participants.^1^


	PARTICIPANT 1			PARTICIPANT 2			PARTICIPANT 3			PARTICIPANT 4			PARTICIPANT 5			PARTICIPANT 6		
					
	PRE	POST	RCI	PRE	POST	RCI	PRE	POST	RCI	PRE	POST	RCI	PRE	POST	RCI	PRE	POST	RCI

Self-Report																		

YSR_Intern	18	14	1.05	21	22	–.32	18	8	3.21	8	10	–.64	15	28	–3.41	28	36	–2.1

YSR_Extern	16	16	.00	11	27	–4.35	11	2	2.45	7	7	.00	34	28	1.80	14	17	–.90

CDI	16	15	0.24	30	16	3.39	13	7	1.45	1	/		14	19	–1.21	21	8	3.14

Feel KJ_Ad (SR)	75	136	5.50	88	103	1.35	116	127	.99	158	175	1.53	133	132	–.09	64	71	.63

Feel KJ_Mal (SR)	91	102	–.99	79	86	.63	87	84	.27	91	59	2.89	78	80	–.19	78	89	.99

Caregiver Report																		

CBCL_Intern	28	5	6.04	14	/	/	27	12	6.09	5	/	/	20	15	1.31	12	13	–.26

CBCL_Extern	11	0	3.29	5	/	/	25	8	4.62	11	/	/	26	17	2.69	16	20	–1.20

Feel KJ_Ad (CR)	68	152	7.58	118	/	/	74	137	5.68	105	/	/	94	124	2.71	89	71	–1.62

Feel KJ_Mal(CR)	107	78	2.62	75	/	/	60	83	2.08	82	/	/	93	96	–0.27	93	88	.45


*Note*. RCI > 1.96 = significant change, RCI > 1.30 and < 1.96 = positive trend, Pre = last week of the baseline, Youth Self Report (YSR), Internalizing (Intern), Externalizing (Extern), Child Depression Inventory (CDI), Adaptive Strategies (Feel KJ_Ad), Maladaptive Strategies (Feel KJ_Mal), Self-Report (SR), Child Behavioral Checklist (CBCL), Caregiver Report (CR).

#### Participant 2

At the start of the 7-week baseline period, participant 2 had a score of 52 on the ERSQ-J (see Figure 3b). These scores fluctuated throughout the baseline period, ranging from 39 to 60. The last two weeks of the baseline phase participant 2 had a stable score of 60 (+15%) which decreased to 50 (–20%) post training. Scores per session indicate a fluctuating scoring pattern. From sessions 2 to 4 (i.e., relaxation and emotional awareness) and sessions 6 to 7 (i.e., self-support) a 10% to 22% increase of the ERQQ-J scores was observed. Notably, at session 9, right after the fifth step (i.e., Analyze), a steep decrease was observed. The RCIs indicated that (see Table 4) there was a significant decrease on the CDI. In addition, a positive trend on the Feel-KJ adaptive strategies (SR) was observed. However, a significant deterioration on the YSR externalizing problems was observed. No significant results were found for the Feel-KJ maladaptive strategies (SR), Feel-KJ adaptive and maladaptive strategies (PR), CBCL externalizing problems or YSR internalizing problems.

#### Participant 3

During the 3-week baseline, participant 3 remained close to stable with a score of 61 at the beginning and a score of 57 at the end of baseline (see Figure 3c). At the end of the treatment phase, there was an increase to 63 (+10%). During training the scores were stable and ranging between 53 and 58. In addition, investigating the RCIs (see Table 4), a significant decrease on both the YSR and CBCL for internalizing and externalizing problems and the Feel-KJ maladaptive strategies (CR) was observed. Furthermore, results show a significant increase on the Feel-KJ adaptive strategies (CR). In addition, a marginally significant decrease on the CDI was observed. No significant results were found for the Feel-KJ adaptive and maladaptive strategies (SR).

#### Participant 4

At the start of the 3-week baseline period, participant 4 had a score of 65. This score increased to 84 (+29%) throughout the baseline phase (see Figure 3d). Post training this score decreased again to 60 (–40%). Scores per session indicate a fluctuating pattern. During the training phase, scores decreased around session 2 and 3 (relaxation and emotional awareness) and then increased again with the highest scores in the second phase of the training (i.e., training specific ER strategies). In addition, looking to the RCIs (see Table 4), a significant decrease on the Feel-KJ maladaptive strategies (SR) and a positive trend on the Feel-KJ adaptive strategies (SR) was observed. No significant results were found for the CDI, Feel-KJ adaptive and maladaptive strategies (PR), CBCL and YSR externalizing problems and externalizing problems.

#### Participant 5

At the start of the 3-week baseline, participant 5 had a score of 45 on the ERSQ-J that declined to 19 (–58%) at the end of baseline (see Figure 3e). At the end of the training this score increased back again to 65 (+71%). At the start of the training, scores immediately increased and ranged between 47 and 65 with the highest scores observed around session 4 (i.e., emotional awareness), 6 (i.e., self-support) and 11–12 (i.e., Problem Solving and Wrap-up). Next, RCIs (see Table 4) showed a significant decrease on the CBCL externalizing problems and a significant increase in the Feel-KJ adaptive strategies (CR). However, a significant deterioration on the YSR internalizing problems and a marginally significant deterioration on the YSR internalizing problems was observed. No significant results were found for the CDI, Feel-KJ adaptive and maladaptive strategies (SR), Feel-KJ maladaptive strategies (CR) and CBCL internalizing problems.

#### Participant 6

During the 3-week baseline, participant 6 started with a score of 27 on the ERSQ-J that decreased to 19 (–30%) at the end of baseline (see Figure 3f). At the end of the training this score increased to 56 (+66%). During the first 3 sessions of the training, scores ranged between 19 and 51 and then remained stable throughout the rest of the training. In addition, RCIs (see Table 4) showed a significant decrease on the CDI. However, a significant deterioration on the YSR internalizing problems and a marginally significant deterioration on the Feel-KJ adaptive strategies (CR) was observed. No significant results were found for the Feel-KJ adaptive and maladaptive strategies (SR), Feel-KJ maladaptive strategies (CR) and CBCL internalizing and externalizing problems.

### Group outcomes

Next to the individual outcomes, pretraining and post training group outcomes were investigated (see Table 5). First, regarding internalizing problems, there was an 18% decline of depressive symptoms (CDI) reported by the adolescents. In addition, as reported by the caregivers, a 42% decline in internalizing problems (CBCL) and a 28% decline in externalizing problems was observed (CBCL). Second, ER outcomes show a 24% increase in ER skills (ERSQ-J) and a 17% increase in adaptive ER strategies (Feel-KJ SR) as reported by the adolescents. Similarly, a 32% increase in adaptive ER strategies (Feel-KJ CR) reported by caregivers was detected.

**Table 5:** Primary outcomes at pretreatment and posttreatment for each participants.


	PRE-INTERVENTIONMEAN (SD)	POST-INTERVENTIONMEAN (SD)	WILCOXON *Z*	*P*	EFFECT SIZE	% CHANGE

Self-Report						

YSR_Intern	18.0 (6.60)	19.66 (10.98)	–.52	.60	–0.15	+1%

YSR_Extern	15.50 (9.65)	16.17 (10.41)	.00	1.00	0.00	<1%

CDI	15.83 (9.58)	13 (5.24)	*–1.48*	.14	–0.43	–18%

ERSQ	48.17 (25.32)	59.67 (5.75)	–.94	.35	–0.27	+24%

Feel KJ_Ad (SR)	105.67 (36.28)	124.0 (34.84)	–1.99	.05*	–0.58	+17%

Feel KJ_Mal (SR)	84.0 (6.39)	83.33 (14.08)	–.53	.60	–0.15	<1%

Caregiver Report						

CBCL_Intern	17.67 (9.0)	10.25 (4.57)	–1.46	.14	–0.42	–42%

CBCL_Extern	15.67 (8.38)	11.25 (9.07)	–1.46	.14	–0.42	–28%

Feel KJ_Ad (CR)	91.33 (18.73)	121 (35.24)	–1.46	.14	–0.42	+32%

Feel KJ-Mal (CR)	85.0 (15.40)	86.25 (7.67)	–.37	.72	–0.11	<1%


*Note*. Youth Self Report (YSR), Internalizing (Intern), Externalizing (Extern), Self-Report (SR), Child Depression Inventory (CDI), Child Behavioral Checklist (CBCL), Parent-Report (PR), *r* = .1 = small effect, *r* = .3 = medium effect, *r* = .5 = large effect.

Wilcoxon Signed-Rank Test showed one significant effect for pretraining and post training change on the adaptive ER strategies (Feel-KJ SR). However, in addition, some notable effect sizes were observed. More specifically, (1) small effects were found for internalizing problems (YSR) and maladaptive ER strategies (Feel-KJ SR); (2) small-to-medium effects were found for ER skills (ERSQ-J); (3) medium-to-large effects were found for depressive symptoms (CDI), internalizing and externalizing problems (CBCL) and adaptive ER strategies (Feel-KJ SR) and (4) large effects were found for adaptive ER strategies (Feel-KJ CR) (see Table 5).

## Discussion

Given the high comorbidity, relapse and symptom shifts in psychopathology, research on transdiagnostic interventions is a promising avenue (Newman et al., 1996; Waddell et al., 2014; Whitney & Peterson, 2019; Wilson, 2020). One core mechanism underlying up to 75% of all DSM-5 disorders is emotion dysregulation (Kring & Sloan, 2009). Based on the well-evidenced adaptive coping with emotions (ACE) model (Berking & Whitley, 2014) and affect regulation training (ART) in adults (Berking & Lukas, 2015), the 12-session EuREKA training for children and adolescents was developed (EuREKA; Verbeken et al., 2019). Although existing research is promising, there is little to no evidence for the effectiveness of EuREKA in clinical groups of youth (Debeuf et al., 2020; Debeuf et al., 2022; Volkaert et al., 2022). Therefore, the current study investigated the feasibility and effectiveness of EuREKA in six adolescents with internalizing and/or externalizing problems in an inpatient treatment center, using a single case research design, taken into account strengths, limitations and clinical implications.

### Feasibility

With regard to the feasibility outcomes as reported by the adolescents, the weekly session rating scale indicated no substantial treatment barriers. On average, participants evaluated the interactions with the trainer, the materials used (i.e., workbook) and the added value of the sessions (i.e., both in terms of content and personal value) positively. A closer look at the individual scores adds nuance to these general findings and shows that three participants gave more neutral responses on some of the items.

Next, based on the overall session scores, participants were, on average, moderately to very satisfied with each session. Half of the participants reported being very satisfied, one reported moderate satisfaction, and two participants provided mixed evaluations, with scores ranging from ‘not satisfied at all’ to ‘very satisfied’. One possible explanation is tied to the trainers’ feedback, which highlighted the crucial role of group atmosphere in the session’s quality. Another explanation could be the significant influence of the session’s topic on participants’ satisfaction.

The aforementioned feasibility outcomes support the use of individual feedback (i.e., routine outcome measurement), for example through a standardized questionnaire (e.g., Session Rating Scale), to monitor both the therapeutic relationship and content of therapy, in order to maintain or adjust the therapeutic approach as needed. (Campbell & Hemsley, 2009; Lambert et al., 2018).

With regard to the feasibility outcomes as reported by the trainers, feedback during the weekly supervision sessions could be categorized in five overarching themes: (1) treatment integrity and self-efficacy; (2) structural feedback on EuREKA, (3) session specific feedback, (4) external factors, and (5) research related feedback. With regard to ‘treatment integrity and self-efficacy’ trainers reported to have reached all goals and felt capable to use the manualized EuREKA protocol. Second, ‘structural feedback on EuREKA’ showed that session length, the long introduction of each session and homework assignments were seen as treatment barriers. Third, with respect to ‘session specific feedback’, the mindfulness exercises were experienced as too lengthy and overly frequent. Several factors may contribute to this perception, including limited attention spans often associated with psychopathology and comorbidities such as a ADHD (Günther et al., 2011). Additionally, negative attitudes toward psychoeducation and tasks resembling schoolwork, particularly for those with a history of academic difficulties, may play a role. A recent review by Herrera et al. (2023) noted that existing meta-analyses have yet to determine whether, and in what ways, variations in session length and format influence the effectiveness of psychoeducation, highlighting the need for further research in this area. Not surprisingly, with regard to ‘external factors’, difficulties with sustained attention and group atmosphere were reported. Finally, ‘research related feedback’ indicated that while the weekly supervision was perceived as valuable, the weekly questionnaires and rating scales were experienced as overly demanding by the adolescents.

### Effectiveness

Overall, the results on the ERSQ-J (completed weekly) indicate variability in individual responses to EuREKA, with the majority of participants reporting improvements in ER skills. Notably, different response patterns emerged: some participants demonstrated stable improvements from the start of the training, while others exhibited more fluctuating trajectories, with key changes occurring in the second phase, after acquiring general ER skills. However, while four out of six participants showed an increase in ERSQ-J scores from pre- to posttraining, two participants reported a decline. These individual variations should be interpreted with caution. For instance, one participant with an initially high baseline score had limited room for further improvement, while another showed highly unstable response patterns, complicating interpretation.

Furthermore, within the current heterogeneous group of adolescents with internalizing and/or externalizing problems, different patterns in changes from pre- to posttraining were observed (see Table 6). First, four out of six participants showed decreases in internalizing problems (i.e;, CDI, YSR or CLBL), with adolescent-caregiver agreement in two cases. Nonetheless, one of these participants showed discrepancy between two measurements (i.e;, CDI and YSR). In addition, one participant reported more internalizing problems after completing EuREKA.

**Table 6:** RCI’s for each participant on each outcome.


	PP1	PP2	PP3	PP4	PP5	PP6

YSR_Intern			✔		**X**	**X**

YSR_Extern		**X**	✔		✔	

CDI		✔				✔

Feel-KJ_Ad (SR)	✔			✔		

Feel-KJ_Mal (SR)				✔		

CBCL_Intern	✔	✔	✔			

CBCL_Extern	✔		✔		✔	

Feel-KJ_Ad (CR)	✔		✔		✔	

Feel-KJ_Mal (CR)	✔		✔			


*Note*. ✔ = clinical significant improvement, ✔ = clinical trend improvement; **X** = clinical significant deterioration, **X** = clinical trend deterioration; Youth Self Report (YSR); Internalizing (Intern); Externalizing (Extern); Self-Report (SR); Child Depression Inventory (CDI); Positive Affect (PA); Negative Affect (NA); Child Behavioral Checklist (CBCL), Caregiver Report (CR).

Next, half of the participants showed decreases in externalizing problems with adolescent-caregiver agreement in two cases. Generally, parent-child correspondence is often higher for more overt than covert behaviours (Achenbach et al., 2002; Achenbach et al., 1987), However, this was not the case in the current study. Moreover, one participant reported experiencing more externalizing problems after completing EuREKA. A possible explanation for the deterioration in internalizing or externalizing problems reported by three participants could be that they did not (yet) fully adopt and automatize the learned ER skills *immediately* after treatment.

Lastly, with respect to the overall group scores, a significant pre-post change in adaptive ER strategies, based on self-report, was observed. In addition, several notable effect sizes were observed across measures. Small effects were found for internalizing problems and self-reported maladaptive ER strategies, small-to-medium effects for ER skills, medium-to-large effects for depressive symptoms, internalizing and externalizing problems, self-reported adaptive ER strategies and large effects for caregiver-report adaptive ER strategies.

### Strengths & Limitations

This study is one of the first to investigate the feasibility and effectiveness of a group training on ER in a clinical sample of referred adolescents with internalizing and/or externalizing problems. Although research on ER as a transdiagnostic treatment is mushrooming, most of the existing research has focused on homogeneous groups, mainly targeting anxiety or depression (i.e., internalizing problems), and have not yet included or evaluated the effects on participants with externalizing problems (Carlucci et al., 2021; Ehrenreich-May et al., 2017). Although research suggests that the association between internalizing problems and ER is stronger compared to that between externalizing problems and ER (Hankin, 2008), our study adds to the literature by providing evidence for the association between ER and both internalizing and externalizing problems, (Beauchaine & McNulty, 2013). In addition, given the considerable co-morbidity between internalizing and externalizing problems, addressing both simultaneously is seen as an added value (Krueger & Markon, 2006; Van der Ende et al., 2016). However, since not all strategies are associated with both problems, it is important to include more than one ER strategy or skill and to treat ER as a multicomponent factor (Aldao et al., 2016; Beauchaine & McNulty, 2013).

Next, this research employed two approaches to gain insight into treatment effectiveness: (1) the traditional approach, in which general results of the sample can be used to make generalizations to the population (however, in this case there is only a very small sample), and (2) an idiographic single-case approach in which the variability within a unique participant is observed (Hersen, 1990; Molenaar & Valsiner, 2009). This approach is supported by the American Psychological Association (APA, (Chambless, 1995) and provides complementary insights compared to a traditional RCT approach. The design allows for a thorough investigation of the processes responsible for intervention induced changes, while controlling for potential confounders (e.g., maturation, repeated testing), and also indirectly monitor adverse reactions or notable distress through weekly measurements (i.e., participants) and supervision (i.e., trainers). Therefore, this approach is considered a scientificly valuable method for increasing confidence that change is attributable to the intervention, while also enhancing generalizability beyond the individual cases studied (Hersen, 1990; Hilliard, 1993; Kazdin & Tuma, 1982).

Nonetheless, some limitations have to be taken into account. First, given the small sample size, the heterogeneous group, and mixed results, it is challenging to draw solid conclusions regarding the *general* effectiveness of EuREKA. To strenghten the case, the research should be replicated multiple times (Molenaar & Valsiner, 2009). Adding a simplified (i.e., fewer and shorter questionnaires) standardized single case research protocol to EuREKA could lower the threshold to psychologists to participate in research, which in turn could help build a large data base of single cases to abstract central features of EuREKA. In the next phase, once feasibility and effectiveness have been thoroughly evaluated and the training has been refined based on the generated insights, its effectiveness and efficacy could be further tested through a randomized controlled trial (RCT), which remains the gold standard for evidence-based practice (Chambless & Hollon, 1998). In addition, to obtain insight into long-term post training outcomes, follow-up measurements could provide important information (Kazdin & Weisz, 1998).

Next, particularly within this training, where specific ER strategies are introduced only during the last sessions, there is a possibility that adolescents have not yet internalized these competencies, which could lead to an underestimation of post training effects. Based on the instructional hierarchy for learning new skills (Haring & Eaton, 1978), it can be assumed that participants in the current study completed the acquisition phase of learning (i.e., phase 1: obtaining adequate competency of the content), but did not reach the fluency phase of skill mastery (i.e., phase 2: becoming fluent in the learned skills). The inclusion of multiple booster sessions—sessions delivered at a later time following the completion of training—has been identified as a critical factor in successful skill acquisition. These sessions facilitate the generalization of learned skills to everyday life and contribute positively to the long-term effectiveness of training (e.g., Durlak et al., 2011; Sanchez et al., 2018). For future research, we recommend systematically evaluating the impact of booster sessions by implementing them at various randomized time points to determine the optimal dose-response for this training.

Another notable limitation is the difficulty participants often face in completing homework assignments, as reported in previous research (e.g., Helbig & Fehm, 2004). Homework is considered a crucial component for facilitating the application of learned skills to real-life situations, with both the quantity and quality of homework completion serving as significant predictors of therapeutic success (Mausbach et al., 2010). To address this issue, the present study incorporated video clips and an engaging workbook to increase the appeal of the assignments. However, we recognize that a traditional workbook format may be perceived as outdated and less accessible or engaging compared to a mobile application on participants’ personal smartphones (Tang & Kreindler, 2017). Ongoing research is currently exploring the potential of a mobile application to support the EuREKA training.

Another limitation to adress is the lack of formal assessment of therapist adherence to the protocol, using for example structured measures such as session recordings or fidelity checklists. However, sessions were conducted in accordance with institutional guidelines for psycho-pedagogical support, and no deviations from the protocol were reported during weekly supervision meetings. Additionally, all participants were provided with individual workbooks, which served as an indirect tool to support adherence to the established protocol.

Lastly, with respect to the ERSQ-J questionnaire, some shortcomings can be noticed. The questionnaire was rather extensive (i.e., 28 items) to be filled oud on a weekly basis. Moreover, adolescents were asked to reflect on the past week when answering the questions, making the questionnaire (overly) sensitive to fluctuations. A shorter questionnaire focusing on the general perception of their own ER skills might provide more accurate weekly outcomes in future research. In addition, tracking personal goals and self-efficacy during training could also added value to this single case research design (Warren & Salazar, 2015). To assess this, ecological momentary assessment (EMA) could be used, in which adolescents repeatedly report on their ER skills and personal goals/self-efficacy in real-time outside therapy (Shiffman et al., 2008).

### Clinical implications

First, this study sheds new light on both research and treatment of youth with internalizing and/or externalizing problems. The current approach suggests that it is feasible and beneficial to train ER within (a) a clinical referred heterogeneous group of youth, and (b) those diagnosed with internalizing and/or externalizing problems. The therapist protocol and participants’ workbook, which provide a step-by-step outline of the training, along with the detailed findings of the training in scientific work (i.e., Braet & Berking, 2024; Boelens et al., 2024), can be widely shared through workshops, white papers, and publications. This dissemination will enhance evidence-based practice in the short term and support improved mental health outcomes for adolescents in the long term.

The findings of this study provide valuable insights for future research aimed at developing and refining evidence-based transdiagnostic treatments for adolescents with internalizing and externalizing problems. Key considerations include addressing challenges related to session length, group formats, the inclusion of booster sessions, and enhancing the appeal of homework assignments. Some of these issues are already being explored in ongoing projects. For instance, a new program featuring shorter sessions spread over a longer period, with a particular focus on improving homework compliance, is currently under development. Building on previous research, it is anticipated that integrating eHealth solutions, such as a mobile app, into psychotherapy could significantly enhance homework adherence (Månsson et al., 2013). Especially, when classic offline face-to-face therapy is combined with online components in a blended care approach (Wentzel et al., 2016), the advantages of both treatment modalities are maximized (e.g., Wolters et al., 2017). An APP that supports the EUREKA training in a blended care format, is expected to have a surplus value making the training (and especially the homework) even more attractive and significantly helpful for adolescents with internalizing or externalizing problems.

## Data Accessibility Statement

The raw data supporting the conclusions of this article will be made available by the authors upon reasonable request.

## Footnotes

Since the required information to calculate RCIs regarding the ERSQ is not (yet) available, this was not included within the current study.
